# Plant disease recognition datasets in the age of deep learning: challenges and opportunities

**DOI:** 10.3389/fpls.2024.1452551

**Published:** 2024-09-27

**Authors:** Mingle Xu, Ji-Eun Park, Jaehwan Lee, Jucheng Yang, Sook Yoon

**Affiliations:** ^1^ Department of Electronic Engineering, Core Research Institute of Intelligent Robots, Jeonbuk National University, Jeonju, Republic of Korea; ^2^ College of Artificial Intelligence, Tianjin University of Science and Technology, Tianjin, China; ^3^ Department of Computer Engineering, Mokpo National University, Mokpo, Republic of Korea

**Keywords:** plant disease recognition, deep learning, dataset making, smart agriculture, precision agriculture

## Abstract

Although plant disease recognition has witnessed a significant improvement with deep learning in recent years, a common observation is that current deep learning methods with decent performance tend to suffer in real-world applications. We argue that this illusion essentially comes from the fact that current plant disease recognition datasets cater to deep learning methods and are far from real scenarios. Mitigating this illusion fundamentally requires an interdisciplinary perspective from both plant disease and deep learning, and a core question arises. What are the characteristics of a desired dataset? This paper aims to provide a perspective on this question. First, we present a taxonomy to describe potential plant disease datasets, which provides a bridge between the two research fields. We then give several directions for making future datasets, such as creating challenge-oriented datasets. We believe that our paper will contribute to creating datasets that can help achieve the ultimate objective of deploying deep learning in real-world plant disease recognition applications. To facilitate the community, our project is publicly available at https://github.com/xml94/PPDRD with the information of relevant public datasets.

## 1 Introduction

Having enough food is a basic requirement for human beings. However, more than 600 million people worldwide are estimated to be exposed to hunger in 2030 according to the United Nations. However, many things threaten the availability of food, and plant disease is one of the most essential. It is estimated that $220 billion is lost due to plant disease according to the Food and Agriculture Organization of the United Nations. It is therefore eager to mitigate them, and recognizing plant diseases is a fundamental mission. However, a traditional way is that human experts have to go to the farm to see the plants and then make decisions. This paradigm is expensive and noisy because training experts takes time and many factors play a role in human’s making decisions such as mood and the time taken to complete work (Kahneman et al., 2021).

Deep learning has shown the potential to recognize plant diseases automatically in recent years (Singh et al., 2018; Liu and Wang, 2021; Thakur et al., 2022; Xu et al., 2022b; Salman et al., 2023; Xu, 2023). To access deep learning models, the dataset is one of the most essential considerations (Krishna et al., 2017; Cui and Athey, 2022; Wright and Ma, 2022; Xu et al., 2023b). High-quality training datasets are expected to achieve decent test performance and superior generalization capability. However, plant disease recognition datasets have received relatively less attention in recent years. We argue that it is worth focusing on these datasets for the following reasons.

First, the current datasets exaggerate the performance of existing deep-learning models. From a general computer vision perspective, a common observation is that deep learning models tend to degrade non-trivially when the training and test datasets are not in the same distribution (Arjovsky et al., 2019; Bengio et al., 2021; Cui and Athey, 2022; Corso et al., 2023), known as poor generalization. In the context of plant disease recognition, the ultimate goal is to secure superior performance in the test process. When the test datasets are heterogeneous from the training datasets, the trained models do not have reliable performance when deploying. For example, very recent papers suggested that a model trained with controlled background images degrades in performance when tested with uncontrolled background images (Ahmad et al., 2023; Guth et al., 2023; Wu et al., 2023). Therefore, making reliable TEST datasets to test the performance of the model is a fundamental issue for real-world applications.

Second, high-quality TRAINING datasets for plant disease recognition are relatively difficult to collect as this requires an essential understanding in both the deep learning and agricultural fields. Compared to generic benchmarks in computer vision such as ImageNet (Deng et al., 2009) and COCO (Lin et al., 2014) that are related to daily-available objects, strong domain knowledge about agriculture and plants is required to create a plant disease recognition dataset. Simultaneously, knowledge about deep learning should be involved, such as the challenges related to current deep learning methods and data annotation strategies. For example, data annotation should be compatible with the application’s objective and deep learning methods, as detailed in Section 2.6. Hence, considering dataset characteristics from both deep learning and plant disease perspectives is another essential issue.

To address such issues, this paper aims to enhance the understanding of the creation of datasets, evaluate the reliability of deep learning models in close to real-world scenarios, and further facilitate the deployment of deep learning for plant disease recognition. Our ambitious objective is to deploy deep learning models in real-world applications effectively, efficiently, reliably, and robustly. Our study is inspired by a current paradigm in deep learning, data-centric AI^1^ (Whang et al., 2023; Zha et al., 2023). To our knowledge, this study is the first to address these issues. In this way, our paper is in the PERSPECTIVE style heterogeneous from the REVIEW papers (Liu et al., 2021; Ouhami et al., 2021; Singh et al., 2021; Thakur et al., 2022; Shoaib et al., 2023) with the aim of investigating the deep learning methods used and presenting current datasets. In summary, this study has the two main contributions:

- It proposes an informative taxonomy for plant disease recognition datasets.
- It presents future directions for creating plant disease datasets using deep learning.

## 2 Taxonomy

Using deep learning to recognize plant disease is an interdisciplinary challenge. Such a holistic application should be considered from both perspectives, which is the motivation of this section. As shown in **Table 1**, a taxonomy is proposed. We hope that it will enhance the understanding of the community with the objectives of real-world applications, the collection of suitable training datasets and reliable test datasets, and the deployment of compatible deep learning methods.

**Table 1:** Taxonomy of datasets to recognize plant diseases using deep learning.

Application objective		It can be considered from interest: types of plant and organ; plant environment, such as field and greenhouse; recognition level, such as classification, localization, and quantitation.
Input modality		It covers optical images, video, text, audio, and so on, as well as combinations.
Image acquirement	Optical sensor	Type of sensor to obtain images, including hyper-spectral, multi-spectral, RGB, thermal, and depth images.
	Platform	Place or device to put the optical sensors, including human hand, robot arm, UAV, aircraft, and satellite.
Image variation		Change and visual variation of images within a class, such as background, illumination, and scale. The images belonging to a class in a dataset may have many or few image variations.
Dataset splitting		Strategies to split a collected dataset into training, test, and validation datasets, including random, spatial, and temporal.
Annotation	Existence	Datasets can be categorized into fully, partly, and not annotated groups if every image, part of images, and no part of images are annotated, respectively.
	Correctness	Strategy to make sure that annotations from human experts are correct. In general, annotations introduce bias and noise and voting is an effective yet expensive strategy to reduce them if experts provide annotations individually.
	Level	Annotation level, including image, instance, and pixel level where annotations are given for a holistic image, every instance of disease, and every pixel.

### 2.1 Application objective

In terms of plant disease recognition, different applications may have specific interests, such as the type of plants and organs. For example, some applications focus on one specific crop such as tomatoes (Fuentes et al., 2017b; Xu et al., 2022a) and apples (Thapa et al., 2020) whereas others may consider multiple crops (Hughes et al., 2015; Liu et al., 2021). Similarly, diseases exist in different organs, such as leaves, fruits, and stems.

Moreover, applications require different recognition levels. When diseases appear, one may wonder what it is, referring to classification. Sometimes, multiple abnormal patterns may occur simultaneously, and localizing them individually is beneficial. Specifically, a plant may have more than one unhealthy symptom where the locations give more precise information. Furthermore, some decisions and remedies can be adopted based on their magnitudes, termed quantitation, such as the number of infected leaves and the severity of an unhealthy leaf. To some extent, the complexities of the aforementioned analysis gradually improve. Fortunately, these analyses can be implemented by choosing the appropriate deep learning methods, such as image classification, object detection, and segmentation (Xu et al., 2023a).

Furthermore, plants may grow in either in a controlled environment, such as a greenhouse, or a field. In general, diverse environmental settings suggest differences that should be considered when developing datasets and deep learning methods.

### 2.2 Input modality

To recognize plant diseases, human experts use multiple senses such as vision and smell. In addition, knowledge from other experts and their own experience also provide benefits. In terms of machines equipped with deep learning, similar scenarios exist. Optical images, a type of vision, are one of the most fundamental modalities of information to recognize plant diseases. They can be obtained with different devices and with multiple sub-categories, as described in the next subsection. Videos and time-series images provide additional information compared to images alone. To be more specific, videos can capture visual patterns of plant diseases from different perspectives and distances that can be taken as accumulated observations. In a similar way, time series images resemble the actions of human experts who investigate the transformation of plant diseases over time to make decisions. In addition, texts are also beneficial for semantic information in nature because they are created by human beings. For example, text can depict the characteristics of plant diseases such as color and their temporal changes. Text can describe images such as the location of diseases and their magnitudes of severity (Fuentes et al., 2019; Wang et al., 2022; Cao et al., 2023). Furthermore, text can be replaced with audio to provide human knowledge. More modalities are possible and encouraged, such as smell and other new ones, because new types of sensors can also be employed in the future (Zhang et al., 2023).

### 2.3 Image acquirement

**Figure 1:**
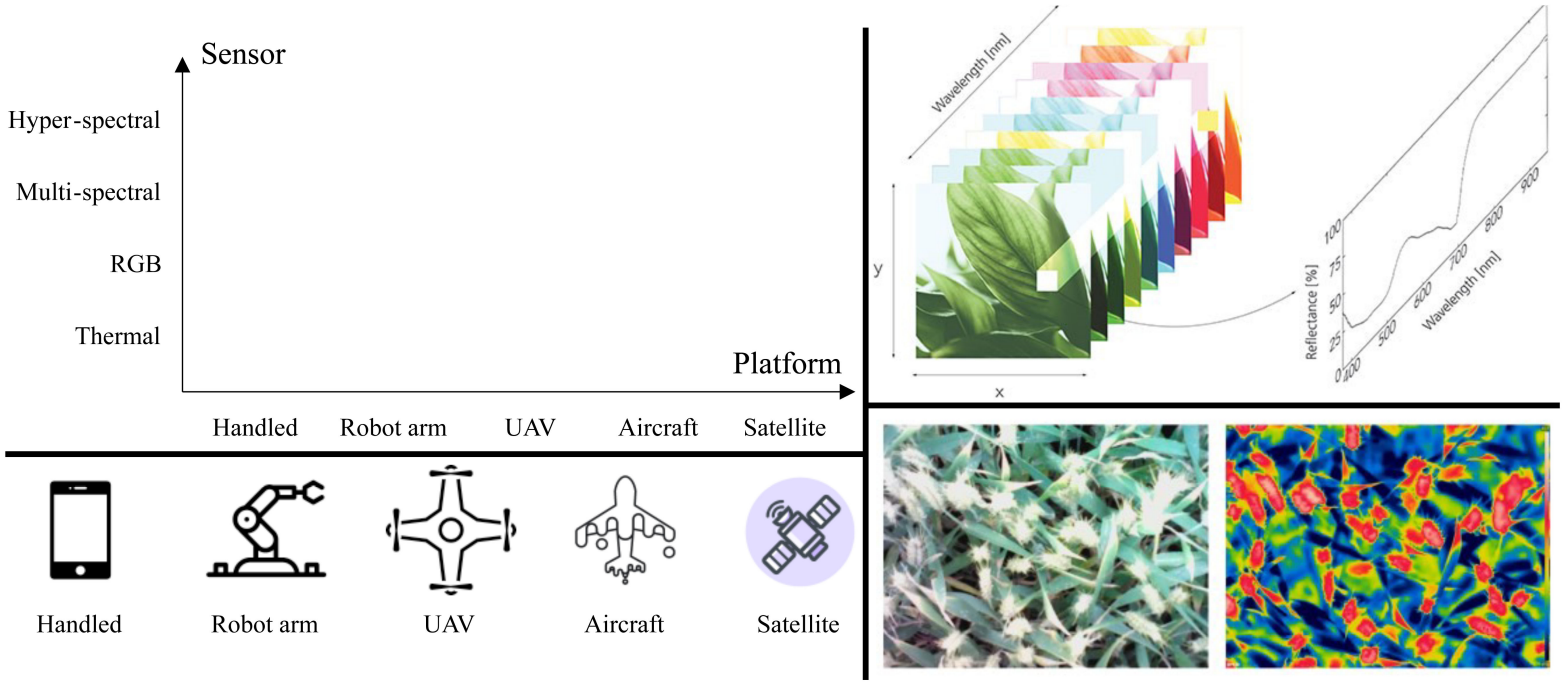
Top-Left: platforms and sensors can be used to obtain plant disease images in different combinations for different purposes such as for plants in fields and greenhouses, adapted from (Xu, 2023) and inspired by (Oerke et al., 2014; Mahlein, 2016). The distances between the plant and platform and the number of channels increase along the horizontal and vertical axes. Further types of sensors and platforms are possible and encouraged. For the advantages and disadvantages of the sensors and platform, please refer to (Oerke et al., 2014; Mahlein, 2016). Bottom-left: visual examples of platforms. Top-right^2^: an illustration of a hyper-spectral image and a multi-spectral image. Bottom-right: an RGB image and the corresponding thermal image using an infrared camera of a wheat canopy (Feng et al., 2021).

Although there are heterogeneous input modalities to recognize plant diseases as mentioned above, optical images are the most widely used. This subsection aims to probe the ways to obtain them since there are multiple types for optical images that are beneficial for diverse cases (Oerke et al., 2014; Mahlein, 2016). As shown in **Figure 1**, optical image acquirement is grouped into two factors, sensors that produce the images and the platform to hold the sensors. This paper focuses on passive sensing, and active remote sensing techniques such as radar are not considered.

The most widely used type of optical sensor is the RGB camera (red, green, and blue) which captures a range of visible wavelengths. Humans understand RGB images very well. One of the reasons for the popularity of RGB images is the great availability resulting from relatively cheap mobile phones. Such phones handled by human beings can produce an enormous number of RGB images. Imagine a scenario where anyone with a mobile phone can take pictures if they are interested in abnormal plants. Furthermore, the resolution is significantly large with clear details. Unlike humans, RGB cameras can be fixed to monitor the growth of plants. RGB cameras placed in robot arms that can move automatically would be an efficient way to free human beings. We argue that this type of image capture is superior to plants in greenhouses. In spite of the super-comfort of RGB images, extra information is also required. For example, thermal sensors are light-free and thus can be employed at night when RGB cameras fail to work. Fluorescence is another possible type although, to the best of our knowledge, there is no related dataset.

The aforementioned sensors take images with a certain range of wavelengths. In contrast, multi-and hyper-spectral sensors can record images with multiple and many ranges of wavelength, resulting in images with many channels. Many vegetation indexes can be obtained with the two sensors (Adão et al., 2017; Lu et al., 2019; Lu et al., 2020; Wan et al., 2022). One of the main advantages is that they can capture images of a large area, beyond a single leaf and plant (Oerke et al., 2014; Mahlein, 2016; Xu, 2023). These two sensors are generally placed in UAVs (unmanned aerial vehicles) and aircraft to surveil many plants. However, their disadvantages are the non-trivial computations resulting from the many channels and being inconvenient to use.

### 2.4 Image variation

In the age of traditional machine learning, engineers and researchers carefully consider data collection, and thus the collected data are relatively small, but informative (Wright and Ma, 2022). This situation has changed in the era of deep learning, where the datasets have become much larger yet non-informative. Sometimes, datasets are collected without any specific objective in advance (Wright and Ma, 2022). Analyzing these datasets is therefore essential, and variation is arguably one of the most important variables for image-based datasets (Fuentes et al., 2017a; Singh et al., 2020; Wu et al., 2023; Xu, 2023; Xu et al., 2023b; Xu et al., 2023a). To achieve decent generalization performance and a basic assumption of machine learning and deep learning, the training and test datasets must be in an identical and independent distribution (i.i.d) (Vapnik, 1991). However, this assumption does not hold in many real-world applications. Hence, we contend that understanding variation within a collected dataset is beneficial for robust applications, and this study focuses on RGB image variation because of its prevalence in recent years.

Officially, image variations consist of inter-class, the diversity between two classes, and intra-class, the diversity within one class (Xu et al., 2023a). One of the basic assumptions in distinguishing plant diseases is that different diseases have different visual patterns even if they are similar (Xu et al., 2023a); otherwise, pattern recognition and classification methods fail. However, recognizing diseases that share some visual patterns, i.e., smaller inter-class image variation, is difficult. Images from one class but with disparate visual patterns, i.e., larger intra-class image variation, such as the flower colors in different growth stages, are also challenging to classify. From the perspective of agriculture, it is inevitable that we have smaller inter-class and larger intra-class image variations. Therefore, deep learning methods are expected to mitigate this challenge. In general, testing models with test images that have similar image variations as the training images tends to lead to high performances. In contrast, deep learning methods are expected to have a poor generalization ability, such that models training only with images from controlled imaging environments will have a low performance when tested with images from uncontrolled ones (Guth et al., 2023; Wu et al., 2023).

**Figure 2:**
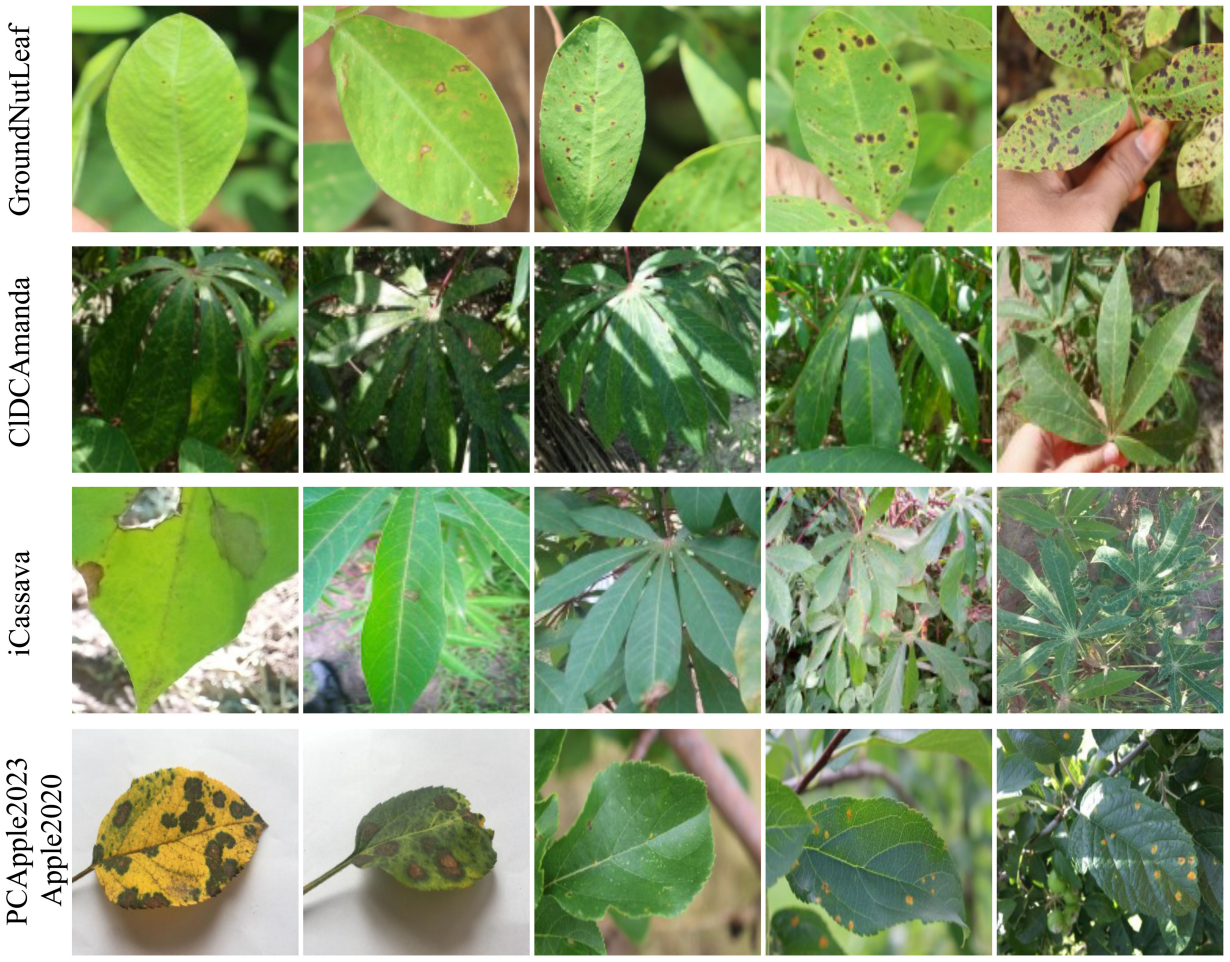
Examples of some image variations from the first to last row: disease stage, illumination, scale, and background. The images are taken from the corresponding datasets. In this paper, the image background is grouped into three groups: simple (the first two images), medium (the third and fourth images), and complex (the last image).

The main image variations are summarized in **Table 2** and **Figure 2** illustrates some image variations. Some variations are closely related. For example, images of the plants in the field may have a much larger diversity in illumination than images from the greenhouse and laboratory. Similarly, canopies tend to have smaller scales than leaves and fruits. Furthermore, additional factors may be the source of multiple variations, for example, a person’s habits when they take pictures could result in diversity in scales and viewpoints. We emphasize that we group backgrounds as either uncontrolled or controlled. For example, leaves are put on homogeneous materials such as paper in the laboratories or field. In the field, plant organs of interest can also be moved to have a simple background. In contrast, with an uncontrolled background, the images are taken without considering the background. Therefore, backgrounds vary significantly and can be controlled when taking pictures of the plants in fields.

**Table 2:** Factors of image variation, partially summarized from (Xu et al., 2023a).

Category	Variation
Plant	Type of plant such as tomato and apple. Plant organs, including leaf, fruit, stem, flower, and canopy. State of plant, such as florescence, and disease, such as with early symptoms. Environment, including field, greenhouse, and laboratory.
Imaging process	Include illumination, scale, viewpoint, and background.

### 2.5 Dataset splitting

In general, three types of datasets are adopted to develop deep learning models. Training datasets are used to train models and validation datasets are used to choose the optimal set of hyper-parameters such as the architecture of the models. After training and validating, test datasets are finally used to check the performance of the trained models. In practical applications, the training and validation datasets are available in the training process, whereas the test dataset is not available until users use the trained model. In such a case, the test performance is not known and thus the model developers cannot assess the trained models. An alternative scheme is to split a holdout dataset as a test before training the models, resembling the real test data. Empirical results suggest that splitting results in a different performance, and thus the splitting strategies should be considered. To be clear, the collected dataset is referred to as the original one that will be split into three parts, training, validation, and test. The real test data from model users are distinguished from the split test dataset.

The most widely used strategy is random splitting. This is when an image in the original data set is randomly placed in one of the three datasets. One of the main issues with this strategy is that multiple images taken for the same observation (e.g., a symptom of plant disease in nature) with only slight differences can be assigned to the training and test datasets, by which the test performance could be overestimated. Moreover, this strategy ignores the generalization challenge (Arjovsky et al., 2019; Bengio et al., 2021; Corso et al., 2023; Xu et al., 2023a), which commonly exists in real-world applications, such that the real test data do not fall into the same distribution as the training dataset.

To mitigate the issues, splitting the data spatially and temporally is appealing. For example, images taken in a place are put into either the training or test datasets (Beery et al., 2022). Similarly, images on the same day or in the same year can be used for only one of the three datasets. In spite of being invariant to the type of plant diseases, the spatial and temporal factors explicitly allow the training and test datasets to be in different distributions. Although the strategy introduces new challenges, such as domain shift (Xu et al., 2023a) as suggested in (Beery et al., 2022), it is worthwhile. Specifically, our objective is to achieve the best performance in the real test process, rather than in the training or the split test datasets. For example, a model trained on the collected data from several farms this year is probably desired to be deployed on different farms for the next couple of years. Beyond spatial and temporal splitting, more things can be considered and encouraged, such as the images being taken by the same person who may have certain habits when taking pictures.

### 2.6 Annotation strategy

To begin with, we explicitly propose the rules for annotating datasets as follows:

- Annotations are difficult, time-consuming, and expensive to obtain.
- Annotations from humans have bias and noise.
- Images and their annotations should simultaneously satisfy the requirements of deep learning methods and agricultural tasks.

The first rule triggers the first question of whether an image is annotated or not, termed the existence of annotation. Usually, all images are fully labeled in related public datasets. On the contrary, all images in a collected dataset are not completely annotated. A more reasonable case is partial annotations where some images are labeled whereas other images are not, mainly because the images are more easily available in a relative manner. In such a scenario, two more factors should be considered: image level, whether an image should be annotated, and class level, how many images should be annotated for a class. We argue that partially annotated datasets should be promoted considering the characteristics of practical applications. In addition, theory also supports it as a marginal distribution of images can be useful with the learned conditional distribution or joint distribution, between labels and images (Bengio et al., 2013).

Furthermore, bias and noise appear when humans make decisions and the magnitude may be underestimated (Kahneman et al., 2021). For example, the validation dataset of ImageNet (Deng et al., 2009), used to perform image classification of generic objects such as dogs and cats, has approximately 6% incorrect labels (Northcutt et al., 2021). Compared to this case, plant disease annotation requires more domain knowledge and it may be more difficult to be precise. For example, three experts had only 85.9% accuracy on average when labeling 999 wheat images (Long et al., 2023). Noisy annotations in the training datasets could result in an unstable training process and inferior test performance, whereas the noise in the validation datasets may lead to the incorrect selection of hyper-parameters (Patrini et al., 2017). Deep learning generally assumes that the annotations are correct and tends to obtain better performance if the annotation noise is smaller (Patrini et al., 2017). Based on this observation, making precise annotations is worthwhile, yet it requires more resources. For example, independent voting by multiple experts tends to be beneficial (Kahneman et al., 2021). In addition, polymerase chain reaction (PCR) may also contribute (Pereira et al., 2023). We emphasize that we are not trying to say that bias and noise should be avoided completely but that they should be noticed when annotating and decreased considering the trade-offs both in the model training and validation stages.

The last law highlights the format of annotation, called levels of the annotation. In general, image classification can be performed at image-level annotation, i.e., an image with a label. Multi-label image classification is also possible if an image contains multiple plant diseases. However, bounding boxes can point out the location of every instance of plant disease in an image and thus is called instance-level annotation. Moreover, pixel-level annotations are desired for the task of segmentation which assigns a label to every pixel. For every level of annotation, extra strategies exist, such as the EEP (Xu et al., 2023a): Exclusion, every annotation includes only one specific visual pattern of plant disease; Extensiveness, every plant disease in the images should have been annotated; Precision, annotation is expected to be precise for different tasks such as the correct labels and precise location of bounding boxes. Again, we highlight that incompatible formats of annotation become feasible with the concept of weak supervision (Zhou, 2018; Xu et al., 2023a) and have a negative impact on test performance. In addition, new types of annotations have emerged and new models may embrace different types of annotation.

**Figure 3:**
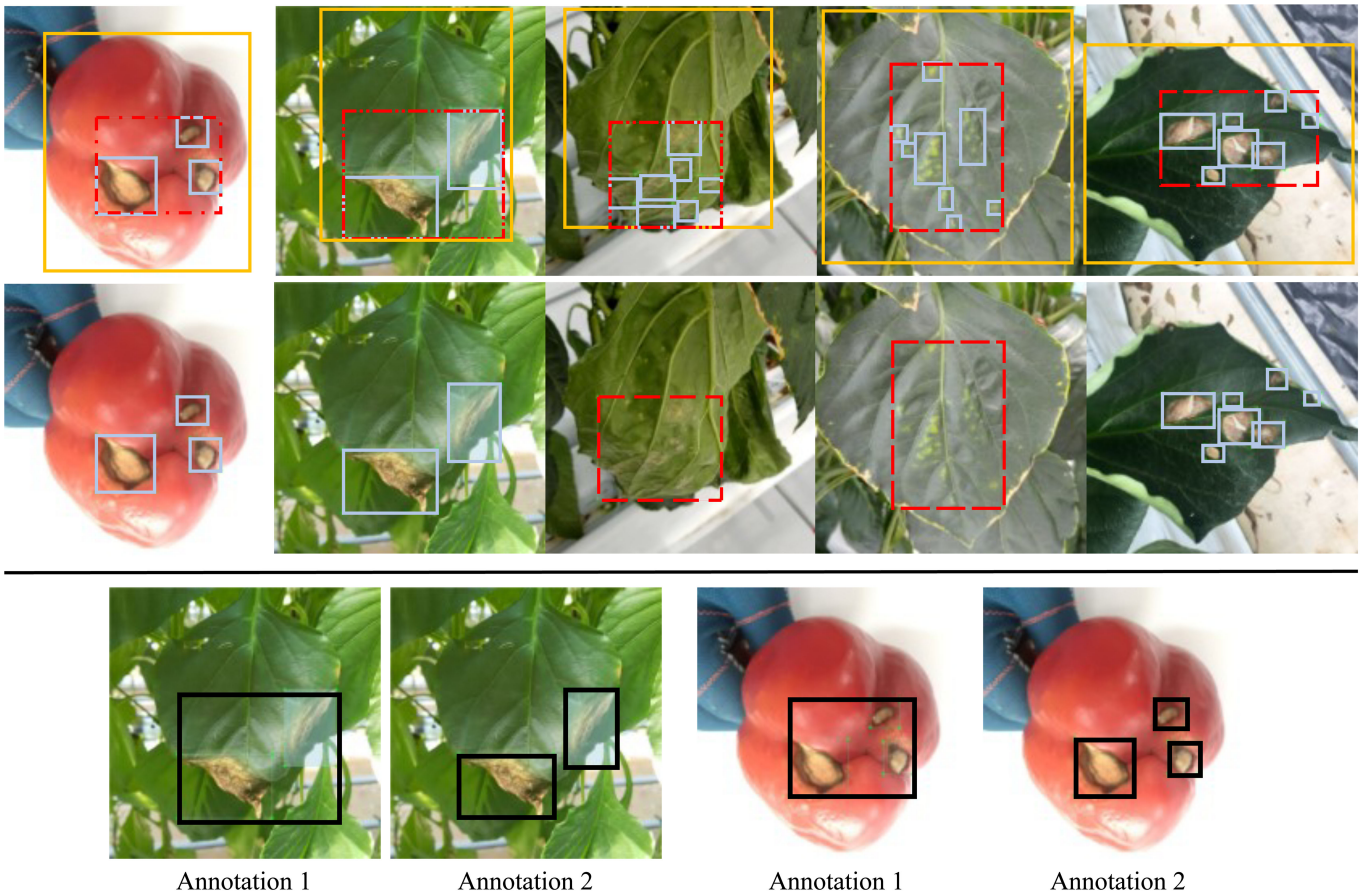
Bounding box annotation strategies in object detection, useful for the localization task. Top row: three strategies for bounding boxes: global (light yellow), which covers one instance such as an instance of fruit or leaf; local (light blue), which covers local areas with dense and intensive symptoms; and semi-global (dotted red), which is a trade-off of previous two, covering local areas yet allowing sparse symptoms such as the case in the first image. Middle row: recommended disease-adaptive strategy in which different diseases may use either local or semi-global strategies. The global strategy is not recommended because an instance may include more than one type of diseases and may include healthy part. Bottom row: inconsistent annotation when the bounding boxes for the same disease are given using different strategies in a dataset, which may confuse the deep learning model and result in optimizing issues. The picture is adapted from (Dong et al., 2022).

Considering the advantages of a localization task using object detection with a weaker image assumption than image classification and a lower annotation workload than segmentation (Xu et al., 2023a), we give more details about it beyond the EEP (Xu et al., 2023a) strategy. To be more specific, how can we give the boundary box for different diseases in a consistent manner, as illustrated in **Figure 3**? In general, three independent strategies can be used for a bounding box. First, every instance of fruit or leaf with diseased symptoms is labeled, termed the global level. The problem is that an instance may have multiple diseases and thus the corresponding bounding boxes will have diverse labels and include the healthy parts, which may confuse deep learning models or cause challenges for model optimization. Second, every single symptom gets a bounding box and the symptom is assumed to be dense without a non-trivial gap, termed the local level. In this case, many bounding boxes may exist in an instance, such as the third and fourth images in the first row of **Figure 3**, which makes annotation harder and more time-consuming. Furthermore, different annotators may have diverse definitions of what constitutes “dense”. Third, the semi-level is a trade-off of the previous two, allowing a gap between symptoms, especially for those that are tiny but many. Based on our understanding and experimental results, an adaptive strategy (Dong et al., 2022) is recommended so that different diseases have different levels of annotation. Another issue is the inconsistency mentioned by Andrew Ng in a video. The underlying issue is that different annotators or even the same annotator at a different time would use different levels of bounding boxes, as shown when comparing annotations 1 and 2 for same image in **Figure 3**. This inconsistency in training datasets gives different information to models, resulting in unstable learning. In addition, inconsistency in the test process may give us an inaccurate evaluation.

## 3 Public plant disease recognition datasets

Based on our preliminary survey, RGB images taken by hand-held cameras dominate in public plant disease recognition datasets. Other types of datasets are rarely utilized or are public. Therefore, we aim to provide a survey on the use of RGB images to recognize plant diseases in this section. We did not do a complete survey, as that is impossible to some extent, and rather focused on the datasets with a relatively higher frequency of utilization or which were released recently, which suggests the tendency in this field. For a dataset, the following tags were considered:

- Dataset name. We will assign a name for the dataset if it was not given one in the original material. The datasets are by default publicly available. Some partial public datasets are also included.
- Plant species. If only one crop is included, the name is given. Otherwise, the number of plant species is given.
- Number of classes. Disease classes and healthy ones are included.
- Number of images. Only the images with publicly available annotations are counted.
- Image background (BG). We split the image background into three categories as shown in the last row in **Figure 2**. The simple one was taken in the environment of a laboratory where the region of interest (RoI) is put on the controlled material. The complex one is taken in the field with a complex background. The medium one is also taken in the field but the RoI may be moved to have a simpler background. Their corresponding abbreviations are sim, med, and cmpx.
- Machine learning (ML) task and official performance (PE). This paper focuses on three types of machine learning tasks as discussed before: image classification (clf), object detection (obj), and segmentation (seg). A dataset may support more than one task. We only report official performance, either in the original publications or from the leaderboard of official challenges. Otherwise, NA denotes not available.

**Table 3** summarizes the related public datasets and our project is publicly available on GitHub with more detailed information. Although we tried to do our best, some beneficial datasets may be not included and thus any new contribution is welcome. One of the main observations of enough descriptions about objectives and usages are lacking. As mentioned above, localization and quantitation analysis are also beneficial, but there are a few relevant datasets with relatively high quality that are available to the public. Another observation is that a decent performance is achieved in most of the reported performance datasets. An exception is that a model trained in the PlantVillage dataset with simple image backgrounds suffers in the FieldPV dataset with different levels of background. A similar situation appears in the FieldPlant dataset. In addition, we found that the majority of the research is unable to use or compare datasets, except the Plant Village dataset. Finally, we point out that, when using the deep learning method, most existing public datasets embrace the i.i.d. assumption ^3^ where the training and test datasets are identical and independent. In this assumption, the original datasets are randomly split into training and test datasets, and thus the reported test performance is more highly estimated than is the case when deploying the trained model.

**Table 3:** Overview of some public plant disease recognition datasets.

Dataset name	Species	Class	Image	Image BG	ML task & PE
Apple2020 (Thapa et al., 2020)	Apple	4	1,821	med	clf: 0.984 AUROC
Apple2021	Apple	6	18,632	med	clf: 0.883 F1
PCApple2023	Apple	9	10,212	med+sim	clf: N.A
ASDID (Bevers et al., 2022)	Soybean	8	9,648	med+sim	clf: 0.968 Acc
BRACOL (Esgario et al., 2020)	Coffee	5	1,747	sim	clf: 0.956 Acc
RoCoLe (Parraga-Alava et al., 2019)	Coffee	6	1,560	med	clf: N.A
iCassava (Mwebaze et al., 2019)	Cassava	5	5,656	med	clf: 0.939 Acc
CLDCMakerere	Cassava	5	21,397	cmpx+med	clf: 0.913 Acc
CLDCAmanda (Ramcharan et al., 2017)	Cassava	6	2,249	med	clf: 0.930 Acc
CLDD	Cassava	3	228	med	clf: N.A
CDRD (Sultana et al., 2023)	Cucumber	8	1,289	med+sim	clf: N.A
CucumberNegm	Cucumber	2	691	med	clf: N.A
PaddyDoctor (Petchiammal et al., 2023)	Rice	10	10,407	cmpx	clf: 0.990 Acc
Rice1426 (Rahman et al., 2020)	Rice	9	1,426	cmpx+med+sim	clf: 0.971 Acc
Rice5932 (Sethy et al., 2020)	Rice	4	5,932	med	clf: 0.984 Acc
HuyDoRice	Rice	4	3,355	sim	clf: 0.984 Acc
DhanShomadhan (Hossain, 2023)	Rice	5	1,106	cmpx+sim	clf: N.A
WheatLong (Long et al., 2023)	Wheat	5	999	cmpx	clf: 0.971 Acc
WheatLeafDataset	Wheat	3	407	med+sim	clf: N.A
GroundNutLeaf (Aishwarya and Reddy, 2023)	Groundnut	5	3,058	med	clf: N.A
MaizeCraze	Corn	6	2,355	sim	clf: N.A
BisqueCorn 1 2	Corn	2	1,785	cmpx	clf: N.A
CornNLB (Wiesner-Hanks et al., 2018)	Corn	1	18,222	cmpx	clf: N.A
iBean	Bean	3	1,296	med	clf: N.A
SoybeanMignoni (Mignoni et al., 2022)	Soybean	3	6,410	cmpx	clf: N.A
TaiwanTomato	Tomato	6	622	med+sim	clf: N.A
GLFD (Rajbongshi et al., 2022)	Guava	5	527	sim	clf: N.A
CitrusRauf	Citrus	10	759	sim	clf: N.A
PlantVillage (Hughes et al., 2015)	14	38	54,305	sim	clf: N.A
FieldPV (Gui et al., 2021)	14	38	665	med+sim	clf: 0.720 Acc
PlantDocCls (Singh et al., 2020)	13	27	2,598	cmpx+med+sim	clf: N.A
PlantConservation (Chouhan et al., 2019)	12	10	4,503	sim	clf: N.A
CCMT (Mensah et al., 2023)	4	22	24,881	med	clf: N.A
PDD271 (Liu et al., 2021)	N.A	271	2,710	cmpx+med	clf: 0.855 Acc
PlantDocObj (Singh et al., 2020)	13	27	2,598	cmpx+med+sim	obj: N.A
NZDLPlantDiseaseV1 (Saleem et al., 2022a)	5	20	3,337	med	obj: 0.745 mAP
NZDLPlantDiseaseV2 (Saleem et al., 2022b)	8	28	3,039	med	obj: 0.932 mAP
FieldPlant (Moupojou et al., 2023)	4	31	5,156	cmpx+med	obj: 0.144 mAP
GrapevineDiseaseMalo	Grape	3	744	cmpx	obj: N.A
GrapevineDiseaseMalo	Grape	4	128	cmpx	seg: N.A
BRACOL (Esgario et al., 2020)	Coffee	2	1,560	sim	seg: N.A
ATLDSD	Apple	5	1,641	med+sim	seg: N.A

Class, image, image BG, ML task, and PE denote the number of classes, number of images, image background, machine learning task, and official performance, respectively. We point out that, when using the deep learning method, most existing public datasets elusively embrace the i.i.d. assumption and the original datasets are randomly split into training and test datasets, which results in a high estimated test performance.

## 4 Future direction of plant disease recognition datasets

- Stage one: verification, where deep learning methods are verified to be useful in recognizing plant diseases.
- Stage two: implementation, where deep learning methods are deployed in real-world applications of plant disease recognition with decent performance.
- Stage three: connection, where plant disease recognition using deep learning methods is connected to downstream applications.

To probe future directions, we first declare three stages of plant disease recognition using deep learning. The first stage is straightforward and almost finished in recent years. However, the second and third stages are still in their infancy. Currently, few publications have mentioned the successful implementation in real-world applications. One of the main reasons for this comes from the assumptions embraced by deep learning methods that generally do not hold in real-world applications. From this perspective, existing datasets accommodated the assumptions. Therefore, one of the future directions of plant disease recognition is to make datasets that violate the assumptions, termed deep learning challenge-oriented datasets. Furthermore, we argue that recognition of plant disease is not the final objective and should be connected with the downstream work, arriving at the third stage. From such a perspective, we argue that another future direction is to make the datasets oriented to downstream applications. Besides achieving better performance in general, two inspirations are discussed, multi-observation and large-scale datasets. These are outlined in the following section with an additional discussion. **Table 4** summarizes our thinking.

**Table 4:** Potential future directions of plant disease recognition datasets.

Deep learning challenge-oriented	Consider the challenges from the perspective of deep learning methods such as learning with noisy data (Dong et al., 2023b), adopting unlabeled inputs (Fang et al., 2021), zero-shot learning (Sun et al., 2024), learning to generalize (Guth et al., 2023; Wu et al., 2023; Xu et al., 2023a), finding and clustering new or unknown diseases (Han et al., 2021), lifelong-learning with iteratively coming input (Dong et al., 2023a), uncertainty quantification and according strategy (Angelopoulos and Bates, 2021; Angelopoulos et al., 2021), and utilization of synthetic data by large models and simulation data from digital twins (Pylianidis et al., 2021)
Application-oriented	Consider the objectives of the applications from the perspective of plant disease recognition such as early symptom classification, similar plant disease recognition, connecting plant disease recognition, and effective and efficient remedy making.
Multi-observation	Consider the skills of human experts to recognize plant disease, such as using different modalities, and compare the difference over time during plant disease (time-series).
Large-scale	Collecting a relatively large-scale dataset with high-quality data, considering the success of a large-language model.
Extra	Making benchmarks for plant disease recognition to develop more powerful models. Supplying metadata and attributes. Datasets analysis. Consider beyond plant disease recognition, such as analyzing the incidence of specific plant diseases and other plant-related tasks.

### 4.1 Deep learning challenge-oriented dataset

Although decent performance is achieved in most datasets, the corresponding trained models may suffer when deploying them in real-world applications. One of the reasons is that the assumptions to achieve good performance are not always valid (Xu et al., 2023a). Violating those assumptions results in challenges when deploying deep learning models. For example, a model trained in the datasets of several farms is desired to give better results when deployed in other farms, termed spatial generalization. In a similar spirit, it is desirable for a model trained in the datasets collected in a particular time duration to be decent when deployed in another time duration, termed temporal generalization. Additional invariant disease characteristics are also expected to have no impacts. However, current datasets do not support this kind of verification. Formally, deep learning challenge-oriented datasets are highlighted to test and develop models for plant disease recognition. Simultaneously, we argue that datasets should have meta-data, such as position and time stamp. In other words, current datasets assume that something in the training and test datasets is shared (Meng et al., 2023). For example, a new plant disease may exist in the testing stage and is desired to be classified from the known classes that exist in the training dataset (Meng et al., 2023), a challenge termed open set recognition. Furthermore, we highlight that realizing the assumptions of deep learning models and incorporating them into the dataset collection stage needs the cooperation of researchers from the agriculture and deep learning fields. Please refer to the detailed challenges regarding the datasets in (Xu et al., 2023a).

### 4.2 Application-oriented dataset

From the perspective of agriculture, recognizing plant diseases may not be the final objective, and downstream work may follow. For example, early visual pattern recognition is beneficial in making some remedies to reduce loss. Therefore, collecting such datasets is appealing. Although some papers aimed to focus on this issue, there is no agreement on the definition of early disease recognition. We contend that such data have two primary characteristics: recognizable patterns and effective remedies. One of the core assumptions embraced by deep learning models is that different plant diseases have their own patterns; otherwise, they cannot be distinguished. Considering that data modalities have heterogeneous advantages and disadvantages, selecting a suitable input modality is essential. However, disease states cause varying degrees of losses and there are difficulties in providing remedies. In an extreme scenario, when a plant disease explodes on a farm and the plants all die, recognizing the corresponding disease is not useful. More applications in the field of agriculture are possible. Although the objectives are from agriculture, we highlight that trade-offs exist such as in the case of early disease recognition which requires the cooperation from engineers in the deep learning field.

### 4.3 Multi-observation dataset

In general, human experts make superior decisions to recognize plant diseases through multiple observations rather than single observations. Inspired by this situation, we contend that deep learning methods can also be improved with multi-observation. Essentially, multiple observations distribute different information. Multi-modal datasets refer to datasets with various modalities for the same plant diseases. For example, given a leaf with a plant disease, various optical images and texts can be made. In addition, datasets can be in a time series, such as taking images of plant diseases at different times. In particular, visual patterns become clearer and easier to recognize when diseases gradually involve. Time-series datasets may mitigate the challenge of the early recognition of plant diseases. For image data, higher test performance can also be due to multi-spatial datasets, such as taking images in different scales and perspectives. For example, some plant diseases have different patterns on the front and back of leaves.

### 4.4 Large-scale dataset

Large-scale datasets tend to be beneficial for model generalization in many general computer vision tasks and datasets (Kaplan et al., 2020; Zhai et al., 2022; Xu et al., 2023c). Therefore, collecting large-scale datasets for plant disease recognition is appealing and worthwhile although it is time-consuming, difficult, and expensive. One way in which this could be done is crowdsourcing (Coletta et al., 2020) by which related people in different locations take images and then upload them to a platform. These images would then be annotated by the community. In this way, the collected datasets have enormous variations and thus contribute to model generalization (Xu et al., 2023a).

### 4.5 Extra discussion

Benchmarks. In recent years, plant disease recognition has witnessed a significant improvement (Singh et al., 2018; Liu and Wang, 2021; Thakur et al., 2022; Salman et al., 2023; Xu et al., 2023a), as well as the number of related publications. However, a relative comparison is relatively lacking to evaluate different models in diverse applications. One of the main reasons is the shortage of benchmarks, i.e., public and widely used high-quality datasets. We argue that this kind of benchmark will facilitate the community and speed up the deployment of deep learning methods in the real-world applications of plant disease recognition.

Meta-data is the information used to describe datasets from different perspectives, usually with tags. Most of the current relevant public datasets only have the types of plant disease. Other types of information are expected to be beneficial, such as spatial and temporary tags. The datasets with meta-data can be used for different applications by making new datasets.

Analysis of datasets. In general, different applications have heterogeneous difficulties and challenges. Datasets show the faces of applications and therefore, analysis of datasets are essential to understand the applications and further to achieve a better performance. However, few datasets have corresponding analysis and one of the expected future research directions is automatic analysis, such as for intra- and inter-class image variations. Furthermore, dataset analysis can be used in an iterative way to make high-quality datasets.

Beyond plant disease recognition. Recognizing plant disease is just one of the fundamental requirements to have decent crop yields. This objective may be further facilitated by incorporating disease recognition and more things. For example, plants may be infected by specific diseases or viruses in some conditions where finding the correlated factors are beneficial to prevent the plants from succumbing to those diseases. In addition, plant disease recognition is plant-related and thus, from a wider perspective, its recognition can be connected to other tasks such as plant species recognition (Xu et al., 2022b; Meng et al., 2023).

## 5 Concluding Remarks

Using deep learning to recognize plant disease is an interdisciplinary challenge and thus requires a unified perspective. Compared to making deep learning models, we highlighted that datasets are also essential if our objective is to deploy models in real-world applications. Making a plant disease recognition dataset reliable and close to real-world applications a requires superior understanding of both the deep learning and agriculture fields. A systematic taxonomy for related datasets was first provided. We specially emphasize dataset splitting and the annotation strategies that are scarcely discussed in the literature and suggest possible challenges in real-world applications. Further, RGB images are observed as the dominant input modality and an extensive summarization was given. Finally, four types of dataset are described as future directions: deep learning challenge-oriented, application-oriented, multi-observation, and large-scale, with an additional discussion.

## Data Availability

The original contributions presented in the study are included in the article/supplementary material. Further inquiries can be directed to the corresponding authors.

## Footnotes

1
https://spectrum.ieee.org/andrew-ng-data-centric-ai.

2
https://www.greenhousecanada.com/expanding-sight-plant-health-beyond-the-naked-eye/.

3
https://www.statisticshowto.com/iid-statistics/.
